# A training program improves poor first aid knowledge and skills among primary school teachers in Ibadan, Nigeria

**DOI:** 10.11604/pamj.2025.50.58.38707

**Published:** 2025-02-24

**Authors:** Abdulmumin Ibrahim, Nadia Adjoa Sam-Agudu, Sadiya Musa Gwadabe, Risikat Eniola Kadir, Boniface Ayanbekongshie Ushie, Adesola Oluwafunmilola Olumide, Oyeyemi Olufemi-Julius Omotade

**Affiliations:** 1 Department of Anatomy, Faculty of Basic Medical Sciences, University of Ilorin, Ilorin, Kwara State, Nigeria,; 2 Institute of Child Health, University of Ibadan, Ibadan, Oyo State, Nigeria,; 3 International Research Center of Excellence, Institute of Human Virology Nigeria, Abuja, Nigeria,; 4 Department of Pediatrics and Child Health, School of Medical Sciences, University of Cape Coast, Cape Coast, Ghana,; 5 Global Pediatrics Program and Division of Infectious Diseases, Department of Pediatrics, University of Minnesota Medical School, Minneapolis, Minnesota, United States of America

**Keywords:** First aid, school teachers, intervention

## Abstract

**Introduction:**

physical injury is a common cause of morbidity and mortality among children. Schools in many resource-limited countries are often not child-protective. We assessed First Aid (FA) knowledge and skills in a cohort of primary school teachers in Ibadan, Nigeria, and we evaluated the effect of a training program on the cohort's FA capacity.

**Methods:**

we randomly selected 70 teachers from 16 primary schools, assigning them to intervention (N=36) and control (N=34). A 26-point survey and simulated scenarios graded on an 18-point scale assessed FA knowledge and skills, respectively. Control teachers received an HIV education talk. We assessed FA knowledge and skill immediately and three months post-intervention. FA knowledge was rated poor (<13), fair (13-17), and good (>17); skills were rated poor (<9), fair (9-11), and good (>11). We used Student t-test/ANOVA and chi-square for continuous and categorical variables, respectively, at p-value < 0.05 level of significance.

**Results:**

no difference in mean FA knowledge between the intervention (7.7 ± 1.9) and control (7.3 ± 2.5) at baseline (p=0.49). Mean baseline FA skills scores between the intervention (2.8 ± 1.8) and control (2.6 ± 2.1) were similar (p=0.59). Compared to the baseline, there was a significant increase in mean FA knowledge immediately (20.3 ± 2.3, p<0.001) and three months post-intervention (18.2 ± 2.0, p<0.001). Mean FA skills scores also increased from baseline, immediately (12.7 ± 1.8, p<0.001), and three months post-intervention (9.6 ± 2.0, p<0.001). There were no significant changes in FA knowledge or skills in the control group.

**Conclusion:**

the training program led to a significantly and short-term sustained improvement in teachers' FA capacity. School teachers can be trained to provide appropriate and timely first aid care for students injured at school.

## Introduction

Childhood injury is a public health problem that requires urgent attention. Children are constantly exposed to hazards and risks in the physical, social, cultural, political, and economic environments in which they grow, making them vulnerable to various types of injury [1]. In the school environment, they are involved in several activities such as physical education, competitive sport, and even when playing, potentially increasing their risk for unintentional injuries. Several studies found a high incidence rate for various types of injuries among children [2-5].

Due to the vulnerability of children to injury, several measures have been put in place to prevent injuries and mitigate the impact of injuries when they occur. These measures include primary, secondary, and tertiary prevention measures instituted before, during, or after the injury event [1]. First aid, a tertiary prevention measure, is “the temporary and immediate care given to a person who is injured or suddenly becomes ill” [6]. It is also administered to preserve life and minimize the consequences of injury and illness until help from a medical practitioner or nurse is obtained [6]. First aid can save a life by preventing medical emergencies that may arise from an injury or illness [7,8]. As children are prone to sustaining injuries in school, teachers need to be able to provide appropriate immediate assistance to them if they sustain injuries such as falls, bites, burns, and other outdoor emergencies. Basic first aid knowledge helps teachers to deal with such emergencies. Teachers should thus learn about different first aid measures that can be implemented in the school to deal with emergencies. Equipping teachers with basic first aid skills will enable them to promptly offer immediate care to the pupils if the need arises.

In developed countries, there are established emergency systems in place that are readily activated when children sustain injuries in school [9]. However, this cannot be said for most low and middle-income countries [9]. Providing teachers with first aid knowledge and skills may effectively reduce the impact of injuries on school-aged children [9]. Several studies have shown an improvement in teachers' first aid knowledge and skills following intervention through education and training [10-14].

School-age children are adventurous. However, they often lack the skills to protect themselves. Hence, this responsibility falls on their parents and teachers [15]. Since school-age children spend most of their time in school, assessing primary school teachers´ first aid knowledge and skills and equipping them with appropriate skills to attend to emergencies can safeguard the lives of children in school and the nation as a whole. We, therefore, assessed primary school teachers' baseline first aid knowledge and skills for an appropriate intervention program. We also evaluated the effect of the intervention, a first aid training program, on the teachers' first aid capacity.

## Methods

**Study design:** we conducted a quasi-experimental study among primary school teachers in Ibadan, Oyo State, in Southwest Nigeria.

**Settings:** the study was conducted in Ibadan between January and June 2014. Ibadan is the third-largest city in Nigeria, with a population of nearly 4 million people, according to the 2006 population census, and the largest by geographical area. Our study setting is Ibadan North Local Government Area (Ibadan North LGA). Ibadan North LGA has the largest concentration of schools in Ibadan, with 188 registered public and private primary schools. The study population was primary school teachers in both public and private primary schools in Ibadan North LGA.

**Participants:** the participants were primary school teachers from both private and public schools in Ibadan North LGA of Oyo State, Nigeria. All teachers in Ibadan North LGA were eligible and had an equal chance of being selected for the study. We used a three-stage sampling method to select 70 primary school teachers from four wards (from a total of 12) and sixteen schools (from a total of 114). We randomized eight schools each into intervention (N=36) and control (N=34) groups using tables of random numbers.

**Variables:** the outcome variables were first aid knowledge and first aid skills. We used a semi-structured interviewer-administered questionnaire to obtain information on teachers´ socio-demographic characteristics, first aid knowledge (with a 26-point non-weighted scale), and first aid skills (using simulated scenarios and graded on an 18-point non-weighted grade sheet). We assessed the teachers´ first aid knowledge and skills scores pre-intervention, immediately post and three months post-intervention. First aid knowledge was rated poor (<13), fair (13-17), and good (>17); skills were rated poor (<9), fair (9-11), and good (>11).

### Data sources/measurements

**Intervention:** the intervention teachers received a day of first aid training which comprised 4 hours of: didactic lectures, practical demonstrations, and skill-building exercises on the initial assessment of injury victims and common school-based injuries. Teachers in the same school were trained on the same day in groups of four to five to enhance participation and ensure participants benefited maximally from the training. The first aid training utilized a modified version of the American Red Cross and Nigeria Red Cross Society training manuals with additional information from the St. Johns Ambulance website [16] and cardiopulmonary resuscitation (CPR)- automated external defibrillator (AED)-First Aid-Participants Manual [17]. We conducted a total of eight training sessions across the intervention schools. We used the same training manual for all eight intervention schools. The Red Cross Society of Nigeria University of Ibadan branch conducted the training, assisted by four trained research assistants and the principal investigator. The knowledge and skills assessment tools (online supplementary file) were adapted from St. John´s Ambulance website [16] and the American CPR-AED-First Aid Participants Manual [17], respectively.

**Control:** we conducted a training workshop on HIV/AIDS transmission and prevention and consideration for HIV/AIDS in the workplace for teachers in the control group. The training comprised didactic lectures, case studies, and discussions, following which we conducted the immediate post-intervention survey. The investigator and an expert on HIV/AIDS education from the University of Ibadan, Department of Health Promotion facilitated the training. We used the same training modules for all eight control schools.

**Assessment and scoring:** we awarded a point for each knowledge question answered correctly and a zero for an incorrect answer. For skills, a point each was awarded for a correctly performed activity and zero for an activity poorly performed. The minimum and maximum obtainable scores were 0 and 26; and 0 and 18 for first aid knowledge and skills, respectively. Three examiners (the investigator, a representative of the Red Cross Society, and one research assistant) independently assessed and scored each participant´s skills using a semi-structured graded sheet. The average score given by the three assessors was computed and awarded to the participants.

Post-intervention phase consisted of the immediate assessment and another assessment done three months after the intervention. The same study instrument used to assess the teachers´ first aid knowledge and skills for the pre-intervention survey was used in the post-intervention phase (immediate and three months post-intervention).

We obtained ethical approval from the UI/UCH Institutional Review Committee (IRC). We obtained consent from the participants after we provided adequate, clear, and complete information about the study.

**Bias:** we used random sampling to select participant teachers and randomized them into control and intervention groups. We prevented the diffusion of information among teachers by not having control and intervention teachers in the same school.

**Study size:** we used the formula for comparison of paired (matched) proportions below to calculate the sample size [18]:


$$N=\frac{\left(Z_{\alpha /2}+Z_{\beta }\right)p\left(1-p\right)}{{\left(p-p_{0}\right)}^{2}}$$

Z_α_= standard normal deviate when the probability of having a type one error, α is 5% = 1.96; Z_α/2_= standard normal deviate when the probability of having a type two error, β is 5% = 1.28; power (1-β) = 90% p = proportion of respondents whose knowledge and skills remain the same after the intervention under the null hypothesis = 50% = 0.5. Previous studies have shown that knowledge and skills increase by 30% on average after intervention [12,13]. Therefore, p_0_= 30% = 0.3. We arrived at N = 20 teachers per group as the minimum required sample size.

**Statistical methods:** we performed all analyses in R statistical software (R Core Team, 2020). We used Student t-test/ANOVA and chi-square for continuous and categorical variables, respectively. Pearson correlations were used to test the association of teachers´ first aid knowledge and skills with teachers´ characteristics. p-values < 0.05 level of significance.

**Ethical considerations:** this study was approved by the University of Ibadan and University College Hospital Ibadan Institutional Review Committee. We obtained consent from eligible teacher participants after we provided complete information about the study and established understanding of the information.

## Results

**General characteristics of the study population:** a total of 70 teachers (36 interventions; mean age ± standard deviation = 44.9 ± 8.4 years; 62 female) participated in this study. Teachers in both arms of the study have similar socio-demographic characteristics (Table 1). The retention rate of the study teachers was 92.9%, with only five participants (three from the intervention and two from the control) not available for the three months post-intervention assessment. Detailed participant characteristics are summarized in Table 1.

**Table 1:** socio-demographic characteristics of participants (teachers) in control and intervention groups recruited from primary schools in Ibadan (N=70)

Socio-demographic characteristics	Control (n=34)	Intervention (n=36)	X^2^ or t-test	p
**Age group (years)**				
30 and less	3 (8.8%)	4 (11.1%)	0.64	0.89
31 - 40	6 (17.6%)	5 (13.9%)		
41 - 50	16 (47.1%)	15 (41.7%)		
51+	9 (26.5%)	12 (33.3%)		
Mean age (±S.D) years	45.0 (±7.62)	44.83 (±9.14)	0.08	0.93
**Sex**				
Male	2 (5.9%)	6 (16.7%)	2.01	0.16
Female	32 (94.1%)	30 (83.3%)		
**Marital status**				
Single	3 (8.9%)	6 (16.7%)	0.96	0.33
Married	31 (91.2%)	30 (83.3%)		
**Number of biological children^1^**				
0	1 (2.9%)	4 (11.4%)	2.04	0.36
1 - 2	6 (17.7%)	7 (20.0%)		
3 - 5	27 (79.4%)	24 (68.6%)		
**Level of education^2^**				
TTC	29 (85.3%)	27 (77.1%)	0.75	0.37
UG/PG	5 (14.7%)	8 (22.9%)		
**Class taught**				
Grade 1-3	18 (52.9%)	13 (36.1%)	2.00	0.16
Grade 4-6	16 (47.1%)	23 (63.9%)		
**Years of teaching^3^**				
10 and less	8 (23.5%)	8 (22.2%)	0.99	0.61
11-20	8 (26.5%)	8 (22.2%)		
21 and above	17 (50.0%)	20 (55.6%)		
Mean (SD) years of teaching	19.1 ± 9.4	19.7 ± 8	0.24	0.81

1 : number of biological children is missing for 1 intervention teacher; ^2^: level of education is missing for 1 intervention teachers; ^3^: years of experience is missing for 1 control teachers; TTC: teacher training certificate; UG: undergraduate degree; PD: postgraduate degree; p≤ 0.05

**Figure 1:**
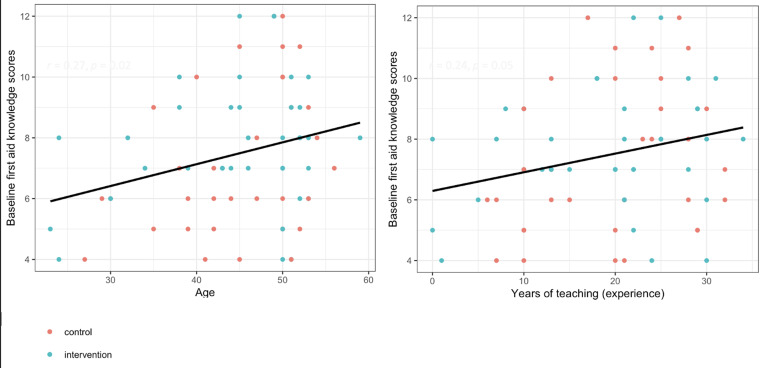
associations of first aid knowledge and skills with teachers´ characteristics

**Baseline first aid knowledge and skills:** we present numbers and percentages of correctly answered first aid knowledge questions and first aid demonstrated skills at baseline. We present numbers and percentages of correctly answered first aid knowledge questions and first aid demonstrated skills at baseline. Teacher participants in both groups answered at least 50% correctly on only 3 out of the 26 first aid knowledge questions (Table 2). Similarly, teacher participants in both groups answered at least 50% correctly on only 3 out of the 18 first aid skill questions (Table 3). Additionally, we present the association of teachers' baseline first aid knowledge and skill scores with age and years of teaching experience. None of the teacher characteristics were associated with their first aid knowledge and skill scores other than their age and years of experience. Increasing age and years of teaching experience were associated with teachers' first aid knowledge and ability to deliver first aid to injured pupils (Figure 1).

**Table 2:** baseline proportion of correct first aid knowledge responses among teachers in control and intervention groups (N=70)

First aid knowledge questions	Control (n=34)	Intervention (n=36)	X^2^	p
First aid? - Help given to a sick/injured person until full medical treatment is available	15 (44.1%)	13 (36.1%)	0.47	0.50
Not an aim of first aid? - Prepare bystanders	6 (17.6%)	10 (27.8%)	1.02	0.31
What should not be in your first aid box? - Antihypertensive medicine	13 (38.2%)	17 (47.2%)	0.58	0.45
Slowing bleeding from a limb? - Elevate the limb above the heart	11 (32.4%)	13 (36.1%)	0.01	0.82
Stuck object in a wound? - Leave it, do not remove	12 (35.9%)	10 (27.8%)	0.01	0.92
Nosebleed? - Can be stopped by briefly pinching the nostril	4 (11.8%)	3 (8.3%)	0.23	0.63
The best position for a child suffering from nosebleeds? - Sat down, leaning forward	5 (14.7%)	6 (16.7%)	0.06	0.82
A serious injury needs immediate attention? - Yes	29 (85.3%)	31 (86.1%)	0.01	0.92
Cuts and scrapes - should not be thoroughly cleaned with soap and water	9 (26.5%)	6 (16.7%)	0.87	0.35
Fracture? - A broken bone	15 (44.1%)	19 (52.8%)	0.04	0.84
Typical symptoms of a fracture? - Immediate excessive swelling, the injured area appears deformed, the farthest point of the injured limb turns blue or is numb to the touch, movement or contact to the injured area causes excessive pain	3 (8.8%)	2 (5.6%)	0.28	0.60
Broken bones - do not always result in compound fracture	8 (23.5%)	8 (22.2%)	0.05	0.82
Ibuprofen - a type of painkiller that also reduces swelling	13 (38.2%)	15 (41.7%)	0.47	0.49
Choking - usually caused by a piece of foreign objects such as food becoming lodged in a person’s esophagus	20 (58.8%)	19 (52.8%)	0.46	0.50
Choking - universal sign is hand to the throat	15 (44.1%)	16 (44.4%)	0.01	0.92
The choking victim - ask whether he/she needs assistance before performing the Heimlich manoeuvre	7 (20.8%)	4 (11.8%)	0.23	0.63
The “five and five” method for choking victim - five back blows and five abdominal thrusts	4 (11.8%)	2 (5.5%)	0.12	0.76
Sprain/strain - rest, immobilize, cold, and elevate	8 (23.5%)	10 (27.7%)	0.05	0.82
CPR - cardio pulmonary resuscitation	2 (5.9%)	3 (8.3%)	0.86	0.35
ABC of CPR - airway, breathing, circulation	19 (0.56%)	16 (44.4%)	0.92	0.34
The first thing to you should do when you find someone collapsed - check for danger	12 (35.3%)	12 (33.3%)	0.70	0.40
Opening a victim's airway - tilt the head backward	6 (17.6%)	8 (22.2%)	0.24	0.74
What to keep checking for once a person is in a recovery position? - Breathing	13 (38.2%)	17 (47.2%)	0.58	0.45
Rate of chest compression to aim for - 100/min	3 (8.8%)	1 (2.8%)	0.94	0.33
How long do you check for breathing before deciding it is absent - 10 secs	7 (20.6%)	9 (25%)	0.02	0.90
When do you stop doing CPR? - When the casualty shows signs of life, when you become exhausted, and when the area/situation becomes too dangerous	22 (64.7%)	21 (58.3%)	0.30	0.60

X^2^: Chi-square; p: significant level at ≤ 0.05

**Table 3:** baseline proficiency in first aid skills among teachers in control and intervention groups (N=70)

First aid skills	Control (n=34)	Intervention (n=36)	X^2^	p
**Initial assessment**				
Assessment of the scene for danger	1 (2.9%)	0	-	-
Assessment of the child’s responsiveness	10 (29.4)	15 (41.7%)	1.14	0.28
Airway	0	0	-	-
Breathing	1 (2.9%)	0	-	-
Circulation	0	0	-	-
Major bleeds (check)	7 (20.6%)	2 (5.6%)	3.53	0.06
**Cardio-pulmonary resuscitation**				
Rescue breath	21 (61.8%)	17 (47.2%)	1.49	0.22
Chest compression	5 (14.7%)	10 (27.8%)	1.78	0.18
Call EMS after five attempts	0	0	-	-
**Choking**				
Ask whether victim is chocked	0	0	-	-
Encourage the victim to cough	0	0	-	-
Perform Heimlich maneuver	0	0	-	-
Call EMS after five attempts	0	0	-	-
**Nosebleed**				
Sit the victim in a comfortable position	19 (55.9%)	24 (66.7%)	0.86	0.35
Lean victim forward	1 (2.9%)	0	-	-
Pinch the victim's nose until the bleeding stops	0	0	-	-
**Fracture**				
Call EMS immediately	3 (8.8%)	5 (13.9%)	0.44	0.51
Immobilized	27 (79.4%)	23 (63.9%)	2.06	0.15

X^2^: Chi-square; p: significant level at ≤ 0.05; EMS: emergency medical services

**Figure 2:**
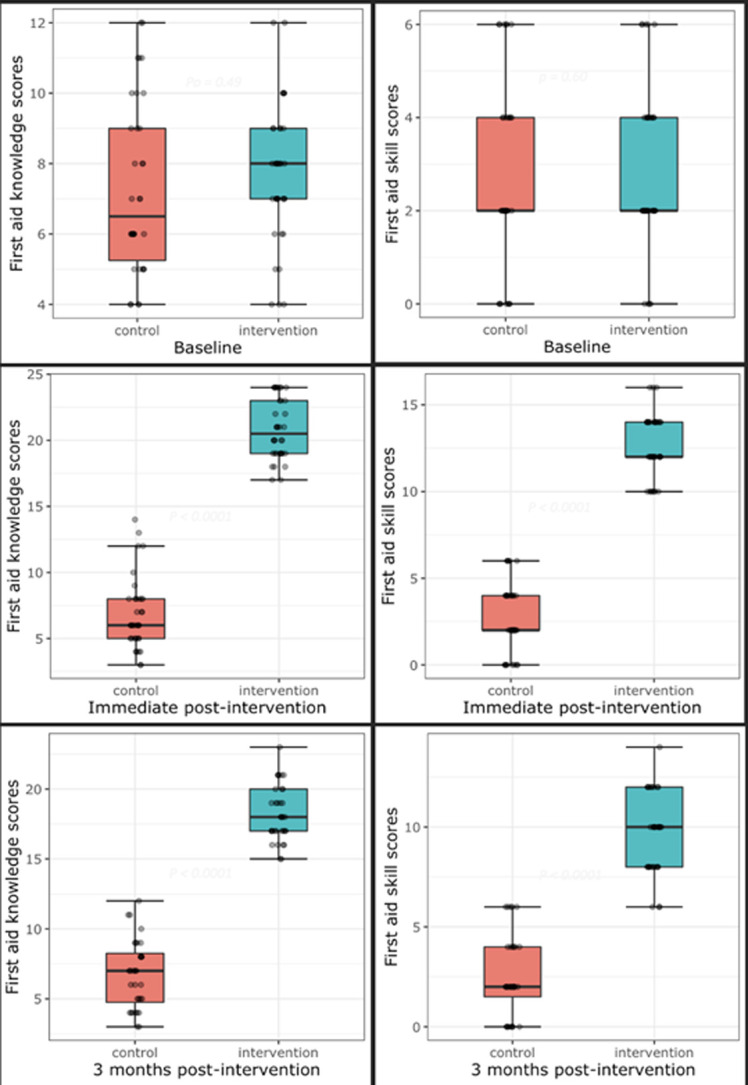
comparisons of first aid knowledge and skill scores across study phases

**Changes in the first aid knowledge and skills:** first aid knowledge and skill scores were comparable between control and intervention teachers at baseline at p = 0.49 and 0.59 for first aid knowledge and skills, respectively. At immediate post-intervention assessment, mean first aid knowledge and skill scores were significantly higher in the intervention teachers compared to controls. Mean first aid knowledge scores (6.9 ± 2.8 control and 20.8 ± 2.3 intervention, p < 0.001); mean skill scores (2.8 ± 1.9 control teachers and 12.7 ± 1.8 intervention teachers, p < 0.001). At three months post-intervention assessment phase, mean first aid knowledge and skill scores were significantly higher in the intervention compared to the control group. Mean first aid knowledge scores were 18.2 ± 2.0 for the intervention and 6.7 ± 2.5 for the control group (p < 0.001). Mean first aid skill scores were 9.6 ± 2.0 and 2.5 ± 2.0 at p < 0.001 for intervention and control, respectively (Figure 2).

**Figure 3:**
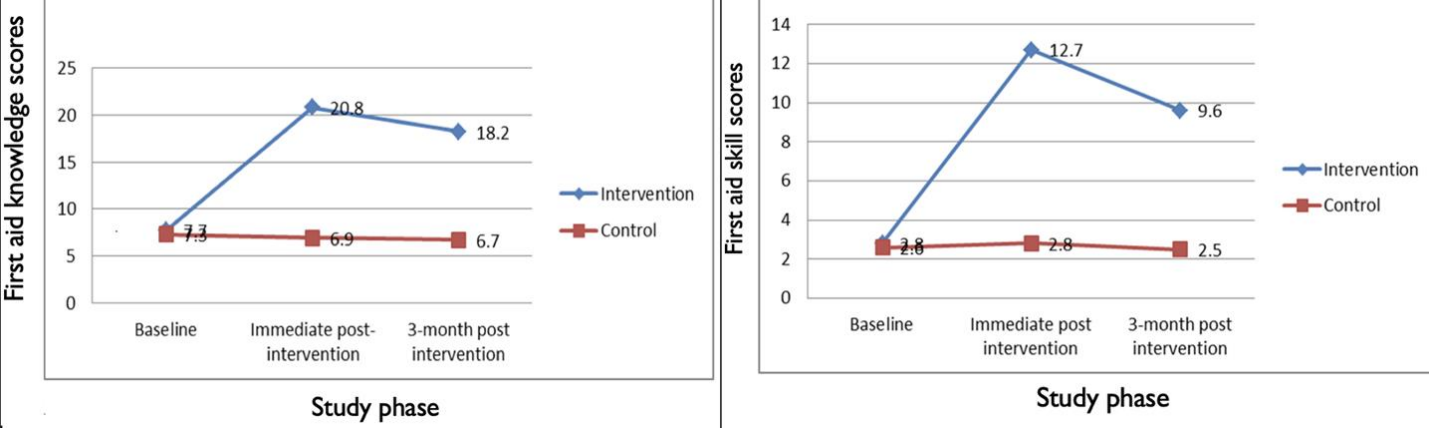
aggregate first aid knowledge and skill score change over the assessment periods

We also determined the change in mean first aid knowledge and skill scores of intervention and control teachers over the three assessment periods. The intervention group had a sharp increase in mean first aid knowledge and skill scores from baseline to the immediate assessment phase, with mean score differences of 13.1 ± 0.4 and 9.9 ± 0.1, respectively. However, the sharp increases declined between the immediate post-intervention and three months post-intervention phases with mean differences of 2.6 ± 0.3 (first aid knowledge scores) and 6 ± 0.3 (first aid skill scores). The first aid knowledge and skills of teachers in the control arm did not change over the three study phases, as observed among the intervention teachers (Figure 3).

## Discussion

Our study aimed to assess the baseline first aid knowledge of teachers and their competency to provide first aid care to children injured at school. Our findings at baseline showed an overall poor mean first aid knowledge among the teachers. More than 90% of teachers had poor first aid knowledge. These findings are not surprising as teachers in resource-rich settings also lack basic first aid knowledge. For example, Gagliardi *et al*. [19] reported that most teachers in the United States had insufficient first aid knowledge of emergency care and basic life support modalities. In Turkey, Baser *et al*. [20] evaluated first aid knowledge and attitudes among primary school teachers and reported a lack of accurate knowledge on how to treat children during medical emergencies. The poor baseline first aid knowledge observed in our study may stem from the fact that first aid training was not a prerequisite in the Nigerian teachers´ training curriculum and has no enforcement by the educational regulatory authority.

The baseline first aid skills of teachers were 100% of teachers were poor. None of the teachers in both arms of the study accurately demonstrated first aid skills using simulated scenarios. Lubrano *et al*. [21] reported that only 8 (1.0%) of the 766 participating teachers had Basic Life Support (BLS) skills at baseline in their study among pre-elementary teachers in the USA. Furthermore, low first aid, BLS, and defibrillation knowledge and skills have been reported among teachers across the world [14,21,22]. Praveen, Mello *et al*., Deutsch *et al*., and Sangowawa *et al*. [10-12,23] independently reported low first aid skill scores at baseline among non-teacher participants. Our study teachers demonstrated poorly performed first aid skills in many first aid skill tasks. Some of the poorly performed tasks include pouring water over pupils who faint, tilting the head of pupils with nosebleeds backward, removing stacked objects from pupils´ bodies, giving rescue breaths to pupils through the nose, and hitting the back of the choked victim. These are bad practices that may have the potential to worsen the condition of the injured victims rather than improve it.

The post-intervention assessments showed improved mean first aid knowledge and skills among the intervention teachers. This finding conformed with results from studies by Sosada *et al*. [22], Ali *et al*. [14], and Patsaki *et al*. [24], who reported improved first aid knowledge and skills among teachers in the intervention group after intervention training. Similarly, Baser *et al*. [20] reported a low (39.9 ± 13.9) mean BLS score before intervention and a high (71.7 ± 17.4) mean BLS score after the Basic Life Support training course among pre-elementary school teachers. It is safe to say that irrespective of teachers' background and socio-economic status, their first aid knowledge and skills can be improved through a well-organized training program.

The intervention teachers retained their first aid knowledge and skills three months after the training. However, there was a slight statistically significant decline in mean first knowledge (20.8 ± 2.3 to 18.2 ± 2.0, p < 0.05) and first aid skills (12.7 ± 1.8 to 9.6 ± 2.3, p < 0.05) scores. This trend was also reported by Sangowawa *et al*. [23] in their study among Nigerian drivers. The slight decline in first aid knowledge and skills might be because our study teachers did not record injuries that require first aid among students in the three-month window period. Therefore, they could not practice the acquired skills suggesting a refresher training may be required to maintain skills gained.

Interestingly, we found an association between first aid knowledge and teachers' age and years of teaching experience. However, age and years of experience did not correlate with the teacher's ability to give appropriate first aid care to injured students. Our finding of no association between teachers' ability to deliver first aid to injured students and teachers' characteristics agrees with the findings by Lubrano *et al*. [21] in Italy.

This study has a few limitations. Firstly, we could not enroll much more than the minimum sample size because it was not feasible to assess and train all teachers in Ibadan, given that the cost of training a teacher was about $50. Therefore, our baseline findings of poor first aid knowledge and skills may not be generalized to Oyo State and Nigeria. Secondly, the study did not consider prospective documentation of injuries in the three-month post-intervention window. Therefore, it is unclear if the decline in knowledge and skills among intervention teachers may not be said to be due to a lack of practice.

## Conclusion

First aid knowledge and skills among primary school teachers in Ibadan were poor at baseline. The training program led to a significant and sustained improvement in teachers' first aid capacity. This study suggests that primary school teachers can be trained to provide appropriate and timely basic care for students injured at school. We, therefore, recommend that education regulatory authorities and policymakers in Nigeria and similar resource-limited settings institute policies that mandate that teachers undergo compulsory first aid training, with regular refresher training to sustain teachers' first aid capacity. These interventions should be accompanied by monitoring the use of first aid skills by teachers, and concrete efforts to make schools safer for children.

### What is known about this topic

- Children of school age are at high risk of physical injury in school;
- The school environment in many resource-limited settings is often not designed to ensure the safety of children;
- Primary school teachers are largely responsible for the protection and safety of pupils during school hours, but their knowledge and skills to deliver first aid are limited.

### What this study adds

- First aid knowledge and skills of the study teachers in Ibadan were poor at the baseline;
- Teachers' first aid knowledge and skills improved significantly in both the immediate post-intervention and three-month post-intervention periods compared to baseline;
- Teachers´ first aid knowledge and skills dropped slightly between the immediate post-intervention and three-month post-intervention phases; this suggests the need for periodic refresher training and monitoring of the use of skills gained.
